# Warm-Up Program for Adolescent Golfers Reduces Low Back Pain: A Double-Blind, Randomized Controlled Trial

**DOI:** 10.1155/tsm2/6993582

**Published:** 2025-08-10

**Authors:** Yuji Hamada, Kiyokazu Akasaka, Yu Okubo, Hiroshi Hattori, Yasuaki Mizoguchi, Yuto Kikuchi, Hotaka Nakagawa, Toby Hall

**Affiliations:** ^1^Saitama Medical University Graduate School of Medicine, Moroyama, Saitama, Japan; ^2^Department of Rehabilitation, Kawagoe Clinic, Saitama Medical University, Kawagoe, Saitama, Japan; ^3^School of Physical Therapy, Saitama Medical University, Moroyama, Saitama, Japan; ^4^Major of Physical Therapy, Department of Rehabilitation, Tokyo University of Technology, Kamata, Tokyo, Japan; ^5^Department of Rehabilitation, Saitama Medical University International Medical Center, Hidaka, Saitama, Japan; ^6^Curtin School of Allied Health, Curtin University, Perth, Western Australia, Australia

**Keywords:** adolescent, athletic injuries, golf, hip, low back pain/prevention & control

## Abstract

**Introduction:** Low back pain (LBP) is common in golfers. While numerous injury prevention programs exist for youth athletes, there is a significant lack of such programs to prevent LBP in adolescent golfers.

**Objective:** To ascertain the impact of an LBP prevention program on the incidence of LBP among adolescent golfers.

**Design:** A double-blind, randomized controlled trial.

**Participants:** Forty-five high-school golfers participated (age, 16.0 ± 0.7 years).

**Interventions:** Participants were randomly allocated to either a Golfer's Low Back Pain Exercise Prevention program (GLEP) group (*n* = 23) or a Sham group (*n* = 22). Both groups were instructed to implement each intervention before playing golf for 12 weeks.

**Outcome Measure(s):** The primary outcomes were the number of people with LBP and number of LBP incidents. Secondary outcomes included the trunk motion direction associated with LBP and the golf swing phase during which LBP occurred. A chi-square test was employed to compare the number of people with LBP. Poisson regression analysis was performed to compare the number of LBP incidents and secondary outcomes between the two groups.

**Results:** There was no significant difference between groups in number of people with LBP over the 12-week period (GLEP: *n* = 5; Sham: *n* = 8; odds ratio = 0.486, *p*=0.279). However, the number of LBP incidents was significantly lower in the GLEP group (GLEP: 16 incidents; Sham: 100 incidents; odds ratio = 0.157, *p* < 0.001). Secondary outcomes showed that the GLEP group had a significantly lower number of LBP incidents during trunk extension and rotation, as well as in all golf swing phases except for the address phase (*p* < 0.05).

**Conclusions:** The GLEP for adolescent golfers over a 12-week period reduced the incidence of LBP. Regular use of GLEP program can be recommended for high-school golfers in LBP prevention.

**Trial Registration:** UMIN Clinical Trials Registry: UMIN000051318

## 1. Introduction

Low back pain (LBP) is a significant global health issue with a reported lifetime prevalence of up to 84% [1]. Due to its high frequency, many individuals perceive it as a transient problem and do not seek medical attention, resulting in a consultation rate of approximately 51% [2]. However, LBP associated with overuse in sports has the potential to indicate underlying serious pathology, which underscores the importance of early detection and intervention [3].

Golf is a sport where injuries are common and frequently affect the lumbar region [4]. In contrast to other sports, golf is a repetitive swing-only sport, so overuse injuries occur more often than traumatic injuries [5]. To our knowledge, several factors have been associated with LBP in golfers, including higher practice frequency, decreased hip range of motion (ROM) [6], decreased trunk muscle strength [7], and differences in muscle activity during the swing [8]. Kim et al. reported that decreased lead hip internal rotation (IR) in golfers significantly increases lumbar rotation during the golf swing [9]. This suggests that if a golfer has restricted hip IR ROM, the load on the lower back is higher.

Dynamic stretching (DS) can increase muscle flexibility and improve ROM [10]. Performing DS on the lead hip external rotator muscles immediately increases hip IR ROM. It has also been reported that increased hip IR ROM is associated with a decrease in lumbar rotation angle [11]. However, as previous studies have not conducted randomized controlled trials of interventions for preventing LBP in golf, further research is warranted in this area. Injury prevention programs are prevalent in other sports as well, with content tailored to the characteristics of the sport [12, 13]. In other words, it is important to develop injury prevention programs specifically for each sport.

The prevalence of LBP during adolescence is high and increases with age [14]. Adolescent athletes experience a greater incidence of LBP than nonathletes [15], and those who experience LBP during adolescence are at an increased risk of LBP in adulthood [16]. One condition that should be considered in adolescent athletes with LBP is lumbar spondylolysis, which may progress to spondylolisthesis if not properly managed [17]. Therefore, it is important to implement measures to address LBP in adolescent athletes. Because LBP is common in golfers, injury prevention is an important issue.

An established and effective method of injury prevention is through a proper warm-up procedure [18, 19]. In golf, players who warm up for more than 10 min have a lower injury rate than those who warm up for less, making appropriate warm-up time crucial [5]. However, 48.3% of golfers do not perform a warm-up, and 38.7% do not consider it necessary [20]. This suggests that warm-up practices may not be sufficiently adopted among golfers.

Developing and implementing appropriate warm-up programs for preventing LBP in adolescent golfers is expected to reduce LBP incidence. Therefore, this study aimed to investigate the effect of a warm-up program on the incidence of LBP among adolescent golfers. If the effect of warming up on golfer LBP can be shown, it could contribute to the field of LBP prevention. We hypothesized that a customized golf-specific warm-up program would decrease the number of LBP episodes.

## 2. Methods

### 2.1. Participants

Participants were recruited between August 2023 and March 2024. To ensure impartiality and account for variations in competition levels, we recruited the top-performing high schools from two prefectures, resulting in a total of 3 schools participating. Research documents were sent to the school principal and club activity advisor. Upon obtaining consent from the school principal and the club advisor, a study briefing was conducted for the participants. All participants provided written consent, and their rights were protected. Inclusion criteria were males and females who were on high-school golf clubs and at least 5 rounds of golf experience. Basic information such as age, height, competition level, and medical history was provided in a questionnaire during the study's introduction phase. Exclusion criteria included individuals who had already experienced LBP and found exercising difficult, as well as players who had taken time off from club activities, and those who did not agree to participate in this study. In accordance with the Declaration of Helsinki, the study was registered in the UMIN Clinical Trials Registry and approved by the Ethics Committee of Saitama Medical University (permit number: 2023-011).

### 2.2. Setup and Protocol

**Figure 1:**
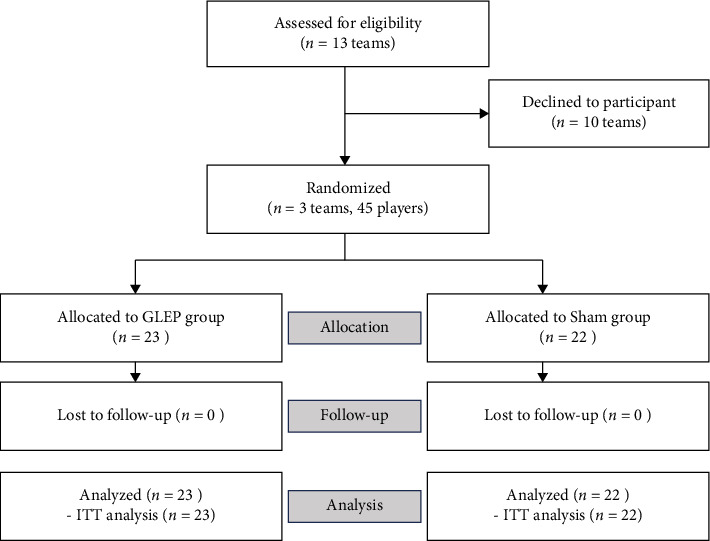
Consolidated Standards of Reporting Trials (CONSORT) flow diagram. GLEP, Golfer's Low Back Pain Exercise Prevention program; Sham, a sham program similar to GLEP. ITT, intention to treat.

This study was a double-blind, randomized, sham-controlled trial (Figure 1, Supporting File 1). Rather than clustering by schools, we opted for individual randomization to mitigate selection bias stemming from varying competition levels across schools. The research collaborator responsible for assigning participants used the Research Randomizer tool to randomly divide the numbers into two groups [13]. No blocking or stratification was used in the randomization process. The combinations of numbers and individuals were made in the order in which they indicated their participation. Researcher blinding was maintained by having only the research collaborator who made the allocation instruct the warm-up routine to the participant. Allocations were strictly controlled and blinded to all except the research collaborator responsible for the assignment during the intervention period. The study had a Sham group, so participants remained unaware of which warm-up method was more effective in preventing LBP. This also maintained blinding for participants. Throughout the study, a blinded researcher made regular visits to the schools every two to 4 weeks. Their role involved encouraging participants to diligently maintain self-report records and inquire about their exercise routines, thereby enhancing adherence to the intervention.

### 2.3. Low Back Pain Prevention Program

**Figure 2:**
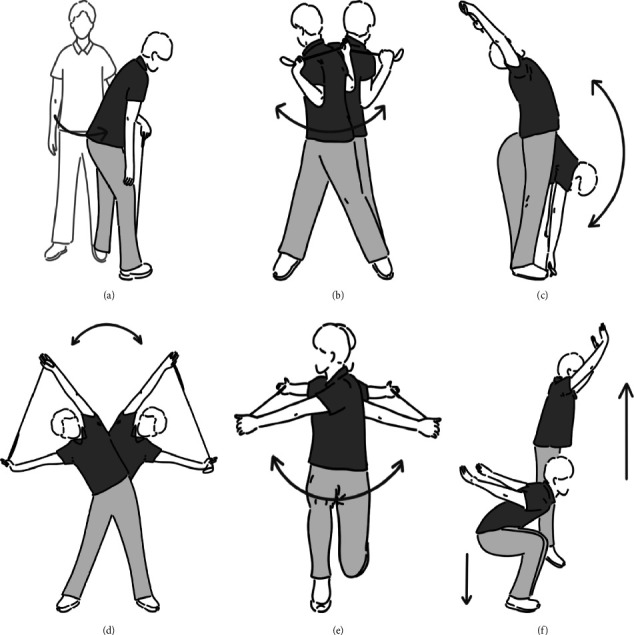
Golfer's Low Back Pain Exercise Prevention program (GLEP): A right-handed golfer is used as an example. (a) Stretch of lead hip external rotator muscles: Rotate the trunk around the lead leg and internally rotate the lead hip joint (10 times). (b) Hip and trunk rotation: Hold the golf club behind the back, feet wider than shoulder width apart, and rotate the hip joints and trunk horizontally. (c) Hip and trunk flexion/extension: Holding a golf club, flex, and extend the hip and trunk. (d) Hip and trunk lateral flexion: With the golf club in both hands and feet held wider than shoulder width apart, perform lateral bending exercises of the trunk. (e) Forward lunges with trunk rotation: Holding the club level with the floor, perform a forward lunge, and then rotate the trunk to the left and right. (b), (c), (d), (e): Both sides, 5 times, total 10 times. (f) Squat jump: Squat until the knee joint is about 90° and jump (5 times).

The intervention was reported in accordance with the Template for Intervention Description and Replication (TIDieR) checklist (Supporting File 2). We devised an exercise program aimed at preventing LBP among golfers named the Golfers' Low Back Pain Exercise Prevention program (GLEP). Research suggests that players' LBP may be associated with limited lead hip IR ROM [6]. Hamada et al. demonstrated that enhancing lead hip IR ROM could potentially reduce lumbar rotation [11]. Hence, the GLEP was formulated with an emphasis on exercises that enhance lead hip IR ROM combined with a proven warm-up regimen beneficial for golfers' performance. It has been recommended to incorporate DS rather than static stretching in golf warm-ups [21]. The GLEP encompasses stretches targeting improvement in lead hip IR ROM, trunk ROM, and the facilitation of trunk movements, including rotation, flexion/extension, and lateral flexion (Figure 2, Supporting File 3).

**Figure 3:**
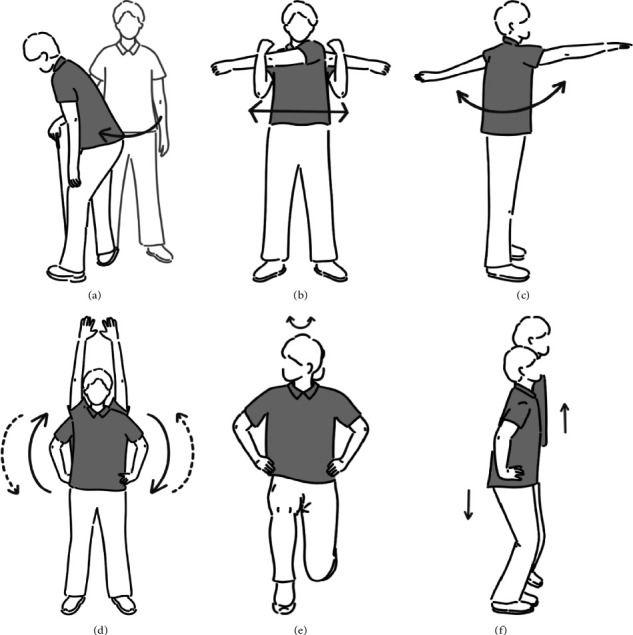
Sham program: A right-handed golfer is used as an example. (a) Stretch of trail hip external rotator muscles: Rotate the trunk around the trail leg and internally rotate the trail hip joint (10 times). (b) Cross-body stretch: Alternating horizontal adduction of the shoulder joint. (c) Shoulder flexion/extension: Flex and extend the shoulder joint to about 90°. (d) Shoulder abduction: Alternate shoulder joint abduction. (e) Forward lunges with neck rotation: Perform a forward lunge and then rotate the neck to the left and right. (b), (c), (d), (e): Both sides, 5 times, total 10 times. (f) Hop exercises: Hop with knee flexed to about 30° and hop (5 times).

The study also devised a sham warm-up to mitigate the Hawthorne effect. This sham warm-up closely resembled the GLEP but was designed to exclude trunk joint movements and lead hip IR. Instead, the sham involved stretching to enhance trail hip IR ROM, followed by cross-body stretch, shoulder flexion/extension, and abduction exercises (Figure 3, Supporting File 4). Like the GLEP, participants were instructed to perform these exercises with maximal joint ROM and without relying on momentum.

Both warm-up protocols remained consistent throughout the study and were supervised directly at the high school's driving range by a therapist with more than 10 years of clinical experience as a physical therapist. Players were segregated into separate rooms during the instruction phase to prevent participants from observing the other group's warm-up. The therapist provided comprehensive explanations of the warm-up procedures using instructional videos, with each group of participants (1–3 individuals) receiving individualized guidance rather than collective instruction. It was explained to the participants that they should not share the instructions they received with each other. Additionally, participants could review the warm-up routine via a video on their cell phones. For blinding purposes, the videos were named “Golfer's Low Back Pain Exercise Prevention Program B” for GLEP and “Golfer's Low Back Pain Exercise Prevention Program A” for the sham. Participants were instructed to independently complete at least two warm-up sets before engaging in golf activities.

### 2.4. Monitoring of Low Back Pain

Participants were surveyed regarding their golf activity, warm-up routines, and LBP experienced during the 12-week study period. Data were collected through Google Forms, with each participant completing the form individually. Golf activity was assessed in terms of practice duration, number of full shots, and number of other full shots. If a participant missed golf due to LBP, the number of days missed was recorded as time-loss days. Regular contact with participants by the club activity advisor emphasized accurately recording self-report information. The location of LBP was determined based on findings from previous studies [15]. If participants reported LBP while playing golf, they were asked to provide details about the pain, including its intensity rated on the Numerical Rating Scale (NRS), ranging from 0 (*no pain*) to 10 (*unbearable pain*). The content of the self-report records was kept to a minimum to reduce participant burden and improve the response rate. Consequently, the type, duration, and extent of pain were not detailed. Participants were also queried about the trunk motion direction inducing pain (flexion, extension, left and right lateral flexion, left and right rotation), the type of golf club used, swing phase (address, backswing, downswing, impact, follow-through, finish), and the number of golf balls hit. Upon completion of data collection, the researcher (YH) was informed of the participants' allocation and proceeded with data processing. The number of people with LBP is the number of people who complained of LBP, while the number of LBP incidents is the total number of times the participant experienced LBP during the period. We understand that many golfers have overuse injuries due to cumulative loading and that many people have LBP that does not require a hospital visit. Therefore, we included even minor LBP (NRS 1-2) in our analysis.

### 2.5. Sample Size

The power analysis application G∗Power3.1.9.2 (https://www.gpower.hhu.de/) was used for sample size determination. Based on previous research on the incidence of injury in youth athletes and the effectiveness of prevention programs, the number of LBP incidents in this study and the effectiveness of the intervention were estimated [12, 19]. For the Poisson regression analysis, statistical significance was set at *α* = 0.05 (two tails) with a power (1 − *β*) of 0.80. A sample size of 38 participants was calculated, and to account for an anticipated 15% dropout rate, at least 42 participants were required for the study.

### 2.6. Statistical Analysis

Statistical analysis was conducted using IBM SPSS Statistics for Mac, Version 28.0 (Armonk, NY: IBM Corp, Released 2020). Independent *t*-tests and Mann–Whitney *U*-tests were employed for basic data comparisons. Descriptive statistics were utilized to calculate golf activity and warm-up adherence over the 12-week assessment period. A chi-square test was employed to compare the number of people with LBP between the two groups. Poisson regression analysis was performed to assess the relationship between the number of LBP incidents, trunk movement direction provoking LBP, and the swing phase associated with LBP. Summary statistics were calculated for the number of time-loss days due to LBP, the number of LBP incidents per 1000 athlete exposures (AEs) and per 1000 athlete hours (AH), the type of club used at the time of LBP, and the number of balls hit. All analyses were conducted on an intention-to-treat basis. The level of significance for this study was set at 0.05.

## 3. Results

### 3.1. Participant

A total of 45 adolescent golfers from three high schools were approved and randomized into either the GLEP group (*n* = 23) or the Sham group (*n* = 22). All participants completed the 12-week intervention period. The flow of participants through the study and randomization procedure is shown in Figure 1.

### 3.2. Baseline Characteristics

A comparison of participant characteristics revealed no significant differences in basic demographics between the two groups, except for age (*p* < 0.05, Table 1). Although the age variance was marginally significant, with a difference of approximately 6 months, post hoc analysis indicated a weak power of 0.33.

Notably, both groups included 12 participants who had experienced LBP within the past year, accounting for 53.3% of the total. During the 12-week data collection period, a response rate of 94.1% was achieved. Among the respondents, participants reported playing golf on 58.1% of the days.  Table 2 details AEs, AH, and warm-up frequency. The change in adherence to the 12-week warm-up implementation did not decrease over the second half of the study period for both groups (group: adherence; 1/12 weeks [%], GLEP group: 78.1/77.6, Sham group: 81.8/82.7). Furthermore, no adverse events related to the intervention were reported in either group.

### 3.3. Primary Outcome

There was no significant difference in the number of people with LBP between groups (GLEP: 5, Sham: 8; odds ratio = 0.486, 95% CI: 0.130–1.816, *p*=0.279; Table 3). However, the number of LBP incidents was significantly lower in the GLEP group (GLEP: 16 incidents, Sham: 100 incidents; odds ratio = 0.157, 95% CI: 0.092–0.266, *p* < 0.001; Table 3). This corresponds to a reduction of about 84% in the number of LBP incidents in the GLEP group. The absolute rate reduction was 8.03 per 1000 AEs, with 1.48 per 1000 AEs in the GLEP group and 9.51 per 1000 AEs in the Sham group. Similarly, the incidence rate was 0.08 per 1000 AH in the GLEP group and 0.55 in the Sham group.

In the GLEP group, five participants reported 1, 2, 3, 4, and 6 incidents of LBP, respectively. In the Sham group, eight participants reported 3 (*n* = 1), 4 (*n* = 1), 8 (*n* = 2), 12 (*n* = 1), 13 (*n* = 2), and 39 (*n* = 1) incidents, respectively. During the study period, 92.3% of participants who developed LBP had experienced golf-related LBP within the past year.

No time-loss days were reported in the GLEP group, whereas two participants in the Sham group reported missing 1 to 7 days of golf due to LBP.

### 3.4. Secondary Outcome

The GLEP group exhibited a reduction in LBP incidents with specific movements, for trunk extension, and right and left rotation (Table 3). Additionally, regarding LBP occurrence during different swing phases, the GLEP group compared to the Sham group showed reduced incidences in all phases except for category “address” and “unknown.”

The driver was the most commonly used club in both groups when LBP occurred (Table 4). Swings that did not require full shots, such as those using approach clubs and putters, tended to cause less frequent LBP. Additionally, the most frequent range of balls hit before the onset of LBP was 10–30, with the majority of respondents using fewer than 100 balls (Table 4).

## 4. Discussion

This study represents the first attempt to evaluate the efficacy of a LBP prevention program tailored for adolescent golfers. While the effectiveness of injury prevention programs for young baseball and soccer players has been well established [12, 22], research on golfers to enhance performance remains limited, with no prior investigations into the effectiveness of warm-up routines for LBP prevention. Given that the most common injury in golfers is in the lower back, there is a pressing need to develop an injury prevention program [23]. Notably, the sole existing injury prevention program for golfers has yet to undergo efficacy testing [24]. Our study findings revealed that the group receiving the GLEP program experienced a substantially lower number of LBP incidents when compared to the Sham group over the 12-week period. This aligns with outcomes from previous studies on injury prevention programs in other sports [13, 25]. There are a few LBP prevention programs that target adolescent athletes, and previous studies have reported that semicustomized interventions are effective for this population [19]. The findings of this study suggest that even a standardized, nonindividualized warm-up program can serve as an effective preventive approach for LBP among adolescent athletes. Several factors contribute to this observed trend. First, the GLEP program included exercises targeting lead hip IR, crucial for reducing lumbar spine rotation during the golf swing. Research indicates that golfers are most prone to LBP during the impact and follow-through phases, necessitating adequate lead hip IR ROM [6, 26]. Additionally, trunk biomechanics during the golf swing reveal peak pelvic rotation velocity during the downswing, accompanied by maximal shearing force on the lumbar spine between the downswing and follow-through [27, 28]. Kim et al. reported that golfers with a restricted hip IR angle had a significantly increased lumbar rotation angle during the golf swing [9]. Previous studies have shown that stretching the lead hip external rotator muscles immediately increases lead hip IR ROM and decreases lumbar rotation angle during the golf swing [11]. Thus, including lead hip IR exercises has emerged as an important means of preventing LBP in golfers. The GLEP group showed a reduced number of LBP incidence during the golf swing (from backswing to finish), supporting the effectiveness of this method in preventing LBP in different phases of the golf swing. These findings suggest that incorporating lead hip IR exercise into a warm-up program is one of the critical factors in reducing LBP in golf. Moreover, the GLEP group also showed a decrease in LBP during trunk extension and rotation movements, which are potentially stressful on the lumbar region, supporting this idea.

Secondly, the high compliance with the program observed in the GLEP group, with a 73.4% implementation rate, underscores the importance of adherence to preventive measures. Previously, it has been reported that high compliance decreases the frequency of injury more than low compliance [12]. Other investigations of injury prevention typically required participants to perform the program one to two times per week [12, 22]. In contrast, our study required participants to perform the program before every golf activity, which could potentially reduce adherence. Nevertheless, we were able to maintain high adherence throughout the study. The utilization of video-based instructional content likely contributed to this high adherence, as previous studies have highlighted the efficacy of video instructions over traditional paper-based materials, particularly for enhancing LBP management [29]. Compared to paper-based instructional materials, video-based content is a cost and time-efficient method that requires fewer human resources. This method is particularly well-suited to school settings, where providing individualized instruction can be difficult, and it facilitates consistent program implementation. Our finding of high compliance in both groups advocates for the adoption of video-based instructional methods in warm-up and training protocols involving movement [30].

Thirdly, the GLEP program emphasized active hip and trunk movements to maximize ROM, aligning with established evidence supporting exercise therapy for LBP prevention [31]. Studies have reported decreased incidence of LBP with the inclusion of hip and trunk exercises in preventive programs [19]. Notably, the golf swing associated with LBP involves early contraction of erector spinae and external oblique muscles, alongside increased lumbar spine lateral flexion velocity [32]. While this study did not directly analyze these effects via electromyography or movement analysis, it suggests that trunk exercises may have influenced these mechanisms.

Among all participants, 53.3% experienced golf-related LBP within the preceding year, with 92.3% of those experiencing LBP during the study period reporting a history of LBP within the past year. This aligns with previous findings, indicating that individuals with a history of LBP are more susceptible to recurrent episodes [33]. It has been shown that those with a higher incidence of golf injuries practice more frequently and take more full shots [26, 33]. Moreover, participants in our study tended to engage in more frequent practice sessions than previous reports, potentially contributing to the higher frequency of LBP occurrences. Detailed analysis revealed that LBP incidents were most commonly associated with the use of drivers, and clubs necessitating full shots. Notably, while the majority of LBP events occurred after hitting 10–30 balls, a significant proportion of players continued playing despite experiencing LBP, indicating a tendency to play through pain. This information has potential value for golf advisors and trainers, highlighting the need to adjust club usage and ball counts during practice sessions for individuals with LBP. Furthermore, these findings are also important for healthcare professionals involved in the care of patients with LBP. When managing these patients, it is important to encourage active engagement to strengthen the therapeutic relationship [34]. Developing an understanding of preventive strategies, such as the GLEP program, is an important component of providing comprehensive, patient-centered care.

### 4.1. Strength and Limitations

This study is the first double-blind randomized controlled trial to evaluate the effectiveness of a warm-up program specifically designed to prevent LBP in adolescent golfers, and it demonstrated the efficacy of the GLEP intervention. Additionally, rather than employing a traditional control group in this study, we chose to compare a sham to the GLEP [13, 19]. The inclusion of a Sham group most likely minimized the Hawthorne effect and also strengthened the methodological rigor of the study.

Another strength of this study is the incorporation of face-to-face, small-group instruction and video materials, which likely contributed to the high compliance rates among participants. This approach also demonstrates that it is a practical and accessible way to promote compliance in the real world. The study also revealed valuable data on AE, AH, and number of balls among adolescent golfers. We identified the golf swing phases and trunk movement directions associated with the development of LBP. These findings may be useful for understanding training loads and injury prevention strategies in adolescent golfers, as well as the biomechanical mechanisms of LBP.

This study has several limitations that should be considered when interpreting the findings. First, participants were instructed to report their LBP cases using Google Forms on the same day whenever possible, which may minimize recall bias. While self-reported LBP incidents provided valuable insights, this method may have introduced recall bias or underreporting of minor episodes. Future studies should consider integrating clinician-confirmed diagnoses to enhance data reliability. Second, the follow-up period of 12 weeks, while sufficient for observing initial effects, may not capture long-term outcomes and sustainability of the GLEP in preventing LBP. Additionally, the study was conducted in an adolescent age group, which may limit the generalizability of the findings to other age groups. Furthermore, the study did not investigate the impact of seasonal variations and environmental factors, such as temperature and humidity, which could influence the incidence of LBP. Another limitation is the lack of detailed diagnostic evaluations for LBP incidents, as participants did not seek medical consultations, potentially leading to an underreporting of severe cases. The compliance rates, while high, were self-reported and may not accurately reflect actual adherence to the intervention. Moreover, the physical function and biomechanical mechanisms underlying the reduction in LBP were not directly assessed, leaving the specific physiological changes induced by the GLEP unclear. Finally, while the study accounted for the Hawthorne effect by including a Sham group, the possibility of residual confounding cannot be entirely ruled out. It has also been suggested that the Sham intervention may have had a therapeutic effect through psychosocial mechanisms, such as the placebo effect [35]. Therefore, a future three-group comparison of GLEP, Sham, and control groups would more clearly evaluate and rigorously test the effectiveness of prevention strategies. Future research should aim to address these limitations by including objective measures of compliance, longer follow-up periods, broader age ranges, and more comprehensive assessments of physical function and biomechanical changes.

## 5. Conclusion

A 12-week customized warm-up exercise program GLEP reduced the incidence of LBP in adolescent golfers by 84% when compared to a Sham program. Implementing the novel GLEP program for adolescent golfers may be an effective intervention to reduce LBP in golf. This finding suggests that incorporating structured injury prevention programs can be beneficial for young athletes in managing and preventing LBP. Future research should explore long-term effects and broader applications of the program across different age groups and golf.

## Figures and Tables

**Table 1:** Characteristics of participants at baseline in the two warm-up groups.

	GLEP (*n* = 23)	Sham (*n* = 22)	*p* value	Mean difference 95% CI		Effects size
Sex, *n*; male/female	19/4	17/5	0.655	0.165	3.109	0.067
Age, y; median (IQR)	16.0 (16.0–17.0)	16.0 (15.0–16.0)	0.042	NA	NA	0.006
Total years played, y; median (IQR)	4.0 (2.0–8.0)	4.0 (1.0–6.3)	0.697	NA	NA	0.104
Height, cm; mean ± SD	170.1 ± 6.2	169.0 ± 5.9	0.523	−0.048	0.025	0.192
Body weight, kg; median (IQR)	61.3 (54.0–71.3)	63.0 (56.0–67.0)	0.883	NA	NA	0.132
Body mass index, kg/m^2^; median (IQR)	21.3 (18.8–23.1)	21.9 (19.7–24.7)	0.440	NA	NA	0.115
Average scores; median (IQR)	83.4 (76.2–94.4)	86.0 (75.7–100.6)	0.865	NA	NA	0.129
LBP experience due to golf for 1 year, *n*	12	12	0.873	0.282	2.935	0.024

*Note:* Continuous data presented as mean ± SD or median (IQR), and categorical variables. GLEP, Low Back Pain Prevention Program for Golfers; Sham, A sham program similar to GLEP.

Abbreviation: LBP, Low back pain.

**Table 2:** Response rate for 12-week self-reported records, number of golf games played, and percentage of warm-ups performed.

	GLEP	Sham	Total
Golf activity			
Survey response rate for 12 weeks, responses (%)	1805/1932 (93.4)	1750/1848 (94.7)	3555/3780 (94.1)
Athlete exposures (AEs), exposure (exposure/12 weeks (%))	1012/1932 (56.1)	1051/1848 (60.0)	2063/3780 (58.1)
Athlete hour (AH), hour	1722	1829	3551
Number of warm-ups performed prior to golf activity, number (%)	720/1012 (71.1)	779/1051 (74.1)	1499/2063 (72.6)
Number of full shots (number/exposure (%))			
Under 100 balls	680 (67.2)	657 (62.5)	1337 (64.9)
Over 100 balls	331 (32.7)	394 (37.5)	725 (35.1)
Unknown	1 (0.1)	0 (< 0.0)	1 (< 0.0)
Number of other full shots (number/exposure (%))			
Under 100 balls	472 (44.9)	572 (54.4)	1044 (49.7)
Over 100 balls	536 (51.0)	479 (45.6)	1015 (48.3)
Unknown	4 (0.2)	0 (< 0.0)	4 (0.2)

*Note:* GLEP, Golfer's Low Back Pain Exercise Prevention program; Sham, A sham program similar to GLEP.

**Table 3:** The occurrence of low back pain in each group and trunk motion and swing phase in which low back pain was observed.

	GLEP	Sham	*p* value	Odds ratio	Mean difference 95% CI	
No. of people with LBP, *n*	5	8	0.279	0.486	0.130	1.816
No. of LBP incidents, number	16	100	< 0.001	0.157	0.092	0.266
Trunk motion with LBP, number						
Flexion	6	26	0.125	0.503	0.209	1.211
Extension	7	78	< 0.001	0.164	0.076	0.357
Right rotation	1	28	0.007	0.104	0.020	0.542
Left rotation	0	50	0.005	0.018	0.001	0.294
Right lateral flexion	1	11	0.135	0.271	0.049	1.502
Left lateral flexion	1	8	0.265	0.370	0.065	2.121
Unknown	2	0	0.125	10.810	0.516	226.323
Golf swing phase with LBP, number						
Address	3	12	0.385	0.588	0.177	1.952
Backswing	1	42	0.001	0.066	0.013	0.342
Downswing	1	29	0.006	0.100	0.019	0.521
Impact	6	47	0.002	0.262	0.113	0.608
Follow-through	3	62	0.000	0.100	0.033	0.298
Finish	7	54	0.000	0.259	0.118	0.569
Unknown	1	5	0.430	0.487	0.082	2.903

*Note:* GLEP, Low Back Pain Prevention program for Golfers; Sham, A sham program similar to GLEP.

Abbreviation: LBP, low back pain.

**Table 4:** Club used and number of balls when low back pain occurs.

	GLEP	Sham	Total
Clubs used, number			
Driver	7	70	77
Fairway wood or hybrid	4	40	44
Iron	7	51	58
Approach	2	14	16
Putter	3	5	8
Unknown	0	6	6
Number of balls, number			
Under 10 balls	2	12	14
10–30 balls	3	50	53
30–50 balls	5	13	18
50–100 balls	4	8	12
100–200 balls	1	11	12
Over 200 balls	0	0	0
Unknown	1	6	7

*Note:* GLEP, Low Back Pain Prevention Program for Golfers; Sham, A sham program similar to GLEP.

## Data Availability

The data that support the findings of this study are available upon request from the corresponding author. The data are not publicly available due to privacy or ethical restrictions.

## Supporting Information

Supporting Information 1 Supporting File 1. CONSORT Checklist: A completed CONSORT checklist outlining the reporting items for the randomized controlled trial.

Supporting Information 2 Supporting File 2. TIDieR Checklist: Template for Intervention Description and Replication (TIDieR) checklist providing detailed information about the intervention.

Supporting Information 3 Supporting File 3. GLEP Video (Low Back Pain Exercise Prevention program B): A video demonstration of the Golfers' Low Back Pain Exercise Prevention (GLEP) program.

Supporting Information 4 Supporting File 4. Sham Video (Low Back Pain Exercise Prevention program A): A video demonstration of the Sham program.
