# Correction to “Warm‐Up Program for Adolescent Golfers Reduces Low Back Pain: A Double‐Blind, Randomized Controlled Trial”

**DOI:** 10.1155/tsm2/9763731

**Published:** 2026-06-20

Y. Hamada, K. Akasaka, Y. Okubo, et al., “Warm‐Up Program for Adolescent Golfers Reduces Low Back Pain: A Double‐Blind, Randomized Controlled Trial,” *Translational Sports Medicine*, 2025, 6993582, https://doi.org/10.1155/tsm2/6993582.

In the article, there are errors for the data reported in the Results and Discussion sections, as well as Tables 1, 2, and 3.

In Section 3.2 of the Results section:

1. “Although the age variance was marginally significant, with a difference of approximately six months, post hoc analysis indicated a weak power of 0.33.”

Should read:

• “Although the age variance was marginally significant, with a difference of approximately six months, post hoc analysis indicated a weak power of 0.51.”
2. “During the 12 weeks data collection period, a response rate of 94.1% was achieved. Among the respondents, participants reported playing golf on 58.1% of the days.”

Should read:

• “During the 12‐week data collection period, a response rate of 94.0% was achieved. Among the respondents, participants reported playing golf on 54.6% of the days.”

In Section 3.3 of the Results section:

1. “The absolute rate reduction was 8.03 per 1000 AEs, with 1.48 per 1000 AEs in the GLEP group and 9.51 per 1000 AEs in the Sham group. Similarly, the incidence rate was 0.08 per 1000 AH in the GLEP group and 0.55 in the Sham group.”

Should read:

• “The absolute rate reduction was 79.3 per 1000 AE, with 15.8 per 1000 AE in the GLEP group and 95.1 per 1000 AE in the Sham group. Similarly, the incidence rate was 9.29 per 1000 AH in the GLEP group and 54.7 in the Sham group.”

In the Discussion section:

1. “Secondly, the high compliance with the program observed in the GLEP group, with a 73.4% implementation rate, underscores the importance of adherence to preventive measures.”

Should read:

• “Secondly, the high compliance with the program observed in the GLEP group, with a 71.1% implementation rate, underscores the importance of adherence to preventive measures.”

Lastly, there are the following errors in Tables 1, 2, and 3:

1. In Table 1, the effect size values for age, total years played, body weight, body mass index, and average scores are incorrect.
2. In Table 2, the percentage values for the following are incorrect: survey response rate for 12 weeks, athlete exposures (AEs), number of warm‐ups performed prior to golf activity, number of full shots under 100 balls, and number of other full shots under 100 balls, over 100 balls, and unknown.
3. In Table 3, the heading for the last two columns is incorrect and should read “95% CI.”

The correct Tables 1, 2, and 3 are shown below.

**TABLE 1:** Characteristics of participants at baseline in the two warm‐up groups.

	GLEP (*n* = 23)	Sham (*n* = 22)	*p* value	Mean difference 95% CI		Effect size
Sex, *n*; male/female	19/4	17/5	0.655	0.165	3.109	0.067
Age, y; median (IQR)	16.0 (16.0–17.0)	16.0 (15.0–16.0)	0.042	NA	NA	0.303
Total years played, y; median (IQR)	4.0 (2.0–8.0)	4.0 (1.0–6.3)	0.697	NA	NA	0.058
Height, cm; mean ± SD	170.1 ± 6.2	169.0 ± 5.9	0.523	−0.048	0.025	0.192
Body weight, kg; median (IQR)	61.3 (54.0–71.3)	63.0 (56.0–67.0)	0.883	NA	NA	0.022
Body mass index, kg/m^2^; median (IQR)	21.3 (18.8–23.1)	21.9 (19.7–24.7)	0.440	NA	NA	0.108
Average scores; median (IQR)	83.4 (76.2–94.4)	86.0 (75.7–100.6)	0.865	NA	NA	0.025
LBP experience due to golf for 1 year, *n*	12	12	0.873	0.282	2.935	0.024

*Note:* Continuous data are presented as mean ± SD or median (IQR) and categorical variables. GLEP, Low Back Pain Prevention Program for Golfers; Sham, a sham program similar to GLEP.

Abbreviation: LBP, low back pain.

**TABLE 2:** Response rate for 12‐week self‐reported records, number of golf games played, and percentage of warm‐ups performed.

	GLEP	Sham	Total
Golf activity			
Survey response rate for 12 weeks, responses (%)	1805/1932 (93.4)	1750/1848 (94.7)	3555/3780 (94.0)
Athlete exposures (AEs), exposure (exposure/12 weeks (%))	1012/1932 (52.4)	1051/1848 (56.9)	2063/3780 (54.6)
Athlete hour (AH), hour	1722	1829	3551
Number of warm‐ups performed prior to golf activity, number (%)	720/1012 (71.1)	779/1051 (74.1)	1499/2063 (72.7)
Number of full shots (number/exposure (%))			
Under 100 balls	680 (67.2)	657 (62.5)	1337 (64.8)
Over 100 balls	331 (32.7)	394 (37.5)	725 (35.1)
Unknown	1 (0.1)	0 (< 0.0)	1 (< 0.0)
Number of other full shots (number/exposure (%))			
Under 100 balls	472 (46.6)	572 (54.4)	1044 (50.6)
Over 100 balls	536 (53.0)	479 (45.6)	1015 (49.2)
Unknown	4 (0.4)	0 (< 0.0)	4 (0.2)

*Note:* GLEP, Golfer’s Low Back Pain Exercise Prevention program; Sham, a sham program similar to GLEP.

**TABLE 3:** The occurrence of low back pain in each group and trunk motion and swing phase in which low back pain was observed.

	GLEP	Sham	*p* value	Odds ratio	95% CI	
No. of people with LBP, *n*	5	8	0.279	0.486	0.130	1.816
No. of LBP incidents, number	16	100	< 0.001	0.157	0.092	0.266
Trunk motion with LBP, number						
Flexion	6	26	0.125	0.503	0.209	1.211
Extension	7	78	< 0.001	0.164	0.076	0.357
Right rotation	1	28	0.007	0.104	0.020	0.542
Left rotation	0	50	0.005	0.018	0.001	0.294
Right lateral flexion	1	11	0.135	0.271	0.049	1.502
Left lateral flexion	1	8	0.265	0.370	0.065	2.121
Unknown	2	0	0.125	10.810	0.516	226.323
Golf swing phase with LBP, number						
Address	3	12	0.385	0.588	0.177	1.952
Backswing	1	42	0.001	0.066	0.013	0.342
Downswing	1	29	0.006	0.100	0.019	0.521
Impact	6	47	0.002	0.262	0.113	0.608
Follow‐through	3	62	0.000	0.100	0.033	0.298
Finish	7	54	0.000	0.259	0.118	0.569
Unknown	1	5	0.430	0.487	0.082	2.903

*Note:* GLEP, Low Back Pain Prevention Program for Golfers; Sham, a sham program similar to GLEP.

Abbreviation: LBP, low back pain.

We apologize for these errors.

